# Draft genome sequences for six *Staphylococcus aureus* isolates from cystic fibrosis patients in Oklahoma

**DOI:** 10.1128/mra.00833-26

**Published:** 2026-10-19

**Authors:** Rebecca Wilson, Noopur Dasgupta, Chelsea L. Murphy, William C. Starr, Kaylea M. Feldman, Elizabeth Pascual, Erika I. Lutter

**Affiliations:** 1 Department of Microbiology and Molecular Genetics, Oklahoma State University 7618, Stillwater, Oklahoma, USA; 2 High-Performance Computing Center, Oklahoma State University 7618, Stillwater, Oklahoma, USA; University of Pittsburgh School of Medicine, Pittsburgh, Pennsylvania, USA

**Keywords:** *Staphylococcus aureus*, genomes, cystic fibrosis

## Abstract

*Staphylococcus aureus* causes many infections and can be life-threatening when present in the lungs of patients with cystic fibrosis (CF). To expand the current knowledge of *S. aureus* infections in CF lung diseases, we report the genomes of six *S*. *aureus* strains isolated from cystic fibrosis patients in Oklahoma, USA.

## ANNOUNCEMENT

Cystic fibrosis (CF) is a genetic disease characterized by recurrent and chronic lung infections by multiple pathogens, including *Staphylococcus aureus (*1, 2). *S. aureus* infections are seen across all CF patient age groups but are more common, particularly in children (3, 4). However, their incidence decreases with age as other bacteria, such as *Pseudomonas aeruginosa*, become more prevalent. Due to this, there are more frequent pulmonary exacerbations, which increase the risk of antibiotic resistance (5).

Sputum samples from individuals with CF were collected from the Pulmonary and Cystic Fibrosis Clinic at Oklahoma Children’s Hospital in Oklahoma City, OK, USA (IRB exempt). The samples were grown on selective media, including Nutrient agar (NA), incubated at 37°C for 96 h, and resulting microbial growth was preserved as a mixed culture in 10% skim milk at −80°C. Isolates were recovered on the general media, NA, and grown aerobically for 48 h. Single colonies were selected and restreaked onto NA under the same aerobic incubation conditions. Cultures were subsequently used to generate freezer stocks. These isolates originated from the sputum collected from both male and female patients who were not experiencing pulmonary exacerbation at the time of the sample collection.

Freezer stock samples were streaked on Mueller-Hinton Agar plates, and an overnight culture was started from the isolated colonies. After 18 h, genomic DNA was extracted from those isolates using a Zymo Fungal/Bacterial DNA Miniprep Kit (Zymo Research Corporation, Irvine, CA, USA). The concentration and purity of the extracted DNA were evaluated on a NanoDrop OneC Microvolume UV-Vis Spectrophotometer and verified by agarose gel electrophoresis. Samples were submitted for whole-genome sequencing using the Illumina platform to SeqCenter (Pittsburgh, PA, USA). Furthermore, Zymo BIOMICS DNA Miniprep was used for DNA extraction. Illumina DNA Prep Kit (tagmentation followed by PCR amplification) was used for library preparation and sequencing of raw reads, which generated 2 × 151 bp paired-end reads. Quality control and adapter trimming were performed using bcl-convert (v4.1.5), and the short-read assembly was conducted with Unicycler (v 0.4.8) (6, 7). QUAST (v5.0.2) and CheckM (1.2.0) were used for assembly statistics (8, 9). Samples were annotated with Bakta (v1.7.0) (10). Annotation was performed by the National Center for Biotechnology Information (NCBI). Multilocus sequence typing (MLST) of the genomes was performed using mlst (v 2.23.0) (11).

**Fig 1:**
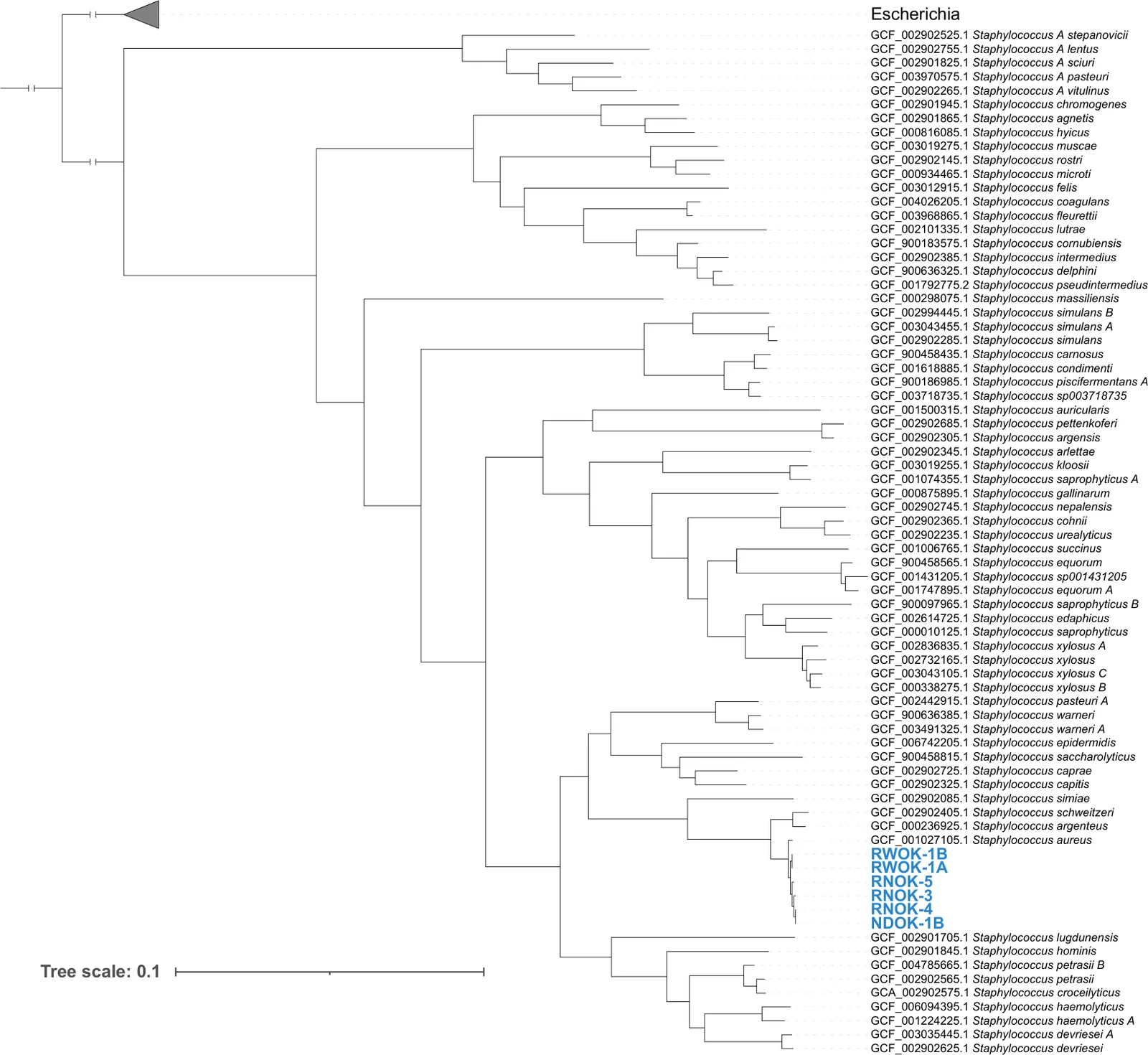
Phylogenetic tree. A maximum-likelihood phylogenetic tree, constructed from 120 single-copy concatenated proteins of the *Staphylococcus* genus. Novel isolates are shown in blue. The tree is rooted using *Escherichia* as an outgroup. Scale represents the average amino acid substitution rate per site.

To verify the taxonomy, a concatenated 120 single-copy protein alignment was generated with GTDB-Tk (v1.3.0) using database r95. *Staphylococcus* and *Staphylococcus A* genomes, along with *Escherichia* outgroup genomes, were extracted, and a maximum-likelihood phylogenetic tree was constructed with FastTree (v2.1.10 SSE3). A concatenated 120 single-copy protein alignment was generated with GTDB-Tk (v1.3.0) (12). *Staphylococcus* and *Staphylococcus A* genomes, along with *Escherichia* outgroup genomes, were extracted, and a maximum-likelihood phylogenetic tree was constructed with FastTree (v2.1.10 SSE3) (13), Fig. 1. The tree was rooted with *Escherichia* and visualized in iTOL (14). Default parameters were used for all programs. The final assembly statistics are mentioned in Table 1.

**TABLE 1:** Genome assembly statistics and accession information for the analyzed bacterial strains

Parameters	Values					
Strain name	RWOK-1A	RWOK-1B	NDOK-1B	RNOK-3	RNOK-4	RNOK-5
Total reads (R1 + R2)	9,244,460	15,453,168	18,509,532	18,470,134	16,589,702	15,682,660
Total size (Mbp)	2.80	2.80	2.82	2.77	2.80	2.84
Coverage	447	719	853	857	767	720
# contigs	38	41	35	39	42	38
N50 (Kbp)	279,593	279,593	222,014	398,562	219,923	224,889
L50	3	3	4	3	4	3
% GC	32.75	32.75	32.78	32.77	32.71	32.77
% Completion	99.51	99.51	99.51	99.23	99.51	99.51
Multilocus sequence type (ST)	ST5	ST5	ST5	ST5	ST5	ST225
NCBI BioSample	SAMN60251906	SAMN60251907	SAMN60251909	SAMN60251910	SAMN60251911	SAMN60251912
NCBI Accession number	JBYMTW000 000000	JBYMTX000 000000	JBYMTZ00 0000000	JBYMUA0 00000000	JBYMUB0 00000000	JBYMUC000 000000
Organism name	*S. aureus* *RWOK-1A 5814FPM1*	*S. aureus* *RWOK-1B 5814FPM5*	*S. aureus* *NDOK-1B 61214B*	*S. aureus* *RNOK-3 6514A*	*S. aureus* *RNOK-4 7314A*	*S. aureus* *RNOK-5 8714C*
NCBI BioProject	PRJNA1467672					

## Data Availability

This Whole Genome Shotgun project has been deposited at DDBJ/ENA/GenBank under the accession nos. JBYMTW000000000, JBYMTX000000000, JBYMTZ000000000, JBYMUA000000000, JBYMUB000000000, JBYMUC000000000. The version described in this paper is JBYMTW010000000, JBYMTX010000000, JBYMTZ010000000, JBYMUA010000000, JBYMUB010000000, and JBYMUC010000000. The raw sequence reads are available in the Sequence Read Archive under the BioProject accession number PRJNA1467672.
